# Associations of overweight and gestational diabetes mellitus with free sugars from solid and liquid sources: cross-sectional and nested case-control analyses

**DOI:** 10.1186/s12889-021-12000-3

**Published:** 2021-10-23

**Authors:** Joseph Mussa, Anne-Sophie Brazeau, Tricia Peters, Mourad Dahhou, Claudia Sanmartin, Nancy Ross, Elham Rahme, Kaberi Dasgupta

**Affiliations:** 1 Department of Medicine, McGill University, Montreal, Quebec, Canada; 2 Centre for Outcomes Research and Evaluation (CORE), Research Institute of the McGill University Health Centre (RI-MUHC), 5252 boul de Maisonneuve Ouest, Office 3E.09, Montreal, QC H4A 3S5 Canada; 3 School of Human Nutrition, McGill University, Montreal, Quebec Canada; 4 Lady Davis Institute of Medical Research, Jewish General Hospital, Montreal, Quebec Canada; 5 Statistics Canada, Division of Health Analysis, Ottawa, Quebec Canada; 6 Department of Geography, McGill University, Montreal, Quebec Canada

**Keywords:** Free sugars, Energy intake, Overweight, Gestational diabetes mellitus, Pregnancy, Sugar-sweetened beverages

## Abstract

**Background:**

Sugar-sweetened beverages have obesogenic and diabetogenic effects ascribed to free sugars. These include added sugars and naturally occurring sugars in juices. A meta-analysis indicates that some foods with added sugars are associated with lower type 2 diabetes rates. To expand the evidence relevant to free sugars from solid sources, we examined a young to middle-aged population with respect to overweight and gestational diabetes (GDM) outcomes.

**Methods:**

We studied female participants (12–50 years old) from the 2004–2005 Canadian Community Health Survey 2.2 (CCHS) with data linked to the hospital Discharge Abstract Database (DAD) until 2017, providing 13 years of follow-up. We estimated free sugars by solid and liquid sources from 24-h dietary recalls as percent total energy intake (TE%), and computed body mass index (BMI). We applied ICD-10 diagnostic codes for deliveries and GDM to DAD. We conducted multivariable logistic regression analyses to evaluate associations between free sugars with overweight at baseline (cross-sectional component) and, in those who delivered, with GDM during follow-up (nested case control component). We compared those with consumption above versus below various thresholds of intake for free sugars, considering solid and liquid sources separately (2.TE%, 5TE%, 10TE% and 15TE% thresholds).

**Results:**

Among 6305 participants, 2505 (40%) were overweight, defined as BMI ≥ 85th percentile below 18 years and BMI ≥ 25 kg/m^2^ for adults. Free sugars from solid sources were associated with lower odds of overweight above versus below the 2.5TE% (adjusted odds ratio [adjOR] 0.80, 95%CI 0.70–0.92), 5TE% (adjOR 0.89, 95%CI 0.79–0.99), and 10TE% (adjOR 0.86, 95%CI 0.75–0.97) thresholds. Free sugars from liquid sources were associated with greater odds of overweight across the 2.5TE% (adjOR 1.20, 95%CI 1.07–1.36), 10TE% (adjOR 1.17, 95%CI 1.02–1.34), and 15TE% (adjOR 1.43, 95%CI 1.23–1.67) thresholds. There were 113 cases of GDM among the 1842 women who delivered (6.1%). Free sugars from solid sources were associated with lower odds of GDM above versus below the 5TE% threshold (adjOR 0.56, 95%CI 0.36–0.85).

**Conclusions:**

Our findings support limiting free sugars from liquid sources, given associations with overweight. We did not identify adverse associations of free sugars from solid sources across any of the thresholds examined.

**Supplementary Information:**

The online version contains supplementary material available at 10.1186/s12889-021-12000-3.

## Introduction

A rise in sugar consumption parallels the increased incidence of overweight [1], gestational diabetes mellitus (GDM) [2], and type 2 diabetes mellitus (T2DM) [3] over the past four decades. Overweight is a risk factor for both GDM and T2DM [1], while GDM is associated with a 7-fold higher risk for T2DM among women in the years following pregnancy [4]. Guidelines for the prevention of obesity, cardiovascular disease (CVD), and T2DM recommend limiting intake of added [5, 6] or free sugars [7, 8]. Added sugars consist mostly of sucrose and high-fructose corn syrup [9]. Free sugars include sugars naturally present in honey, syrup, fruit juice and its concentrates, in addition to added sugars [1]. Most of the evidence concerning sugars and health outcomes [2, 10–13] is driven by sugar-sweetened beverages (SSB), but 60% of free sugars consumed in the United States are from foods [14].

There is debate as to whether any effects of sugars are predicated on their source, namely food or solid versus beverage or liquid source [9]. However, no current guidelines specify source-specific thresholds for intakes of free sugars. The existing guidelines on the intake of sugars specify an upper threshold above which free sugar consumption is not recommended (expressed as a % of total energy [TE] intake, TE%), irrespective of the source [5–8]. The 2015 Dietary Guidelines Advisory Committee (DGAC) advocates limiting added sugars, a subset of free sugars, to 10% of TE intake [5]. The 2015 World Health Organization (WHO) guidelines also suggest a 10% limit but for free sugars in general, and thus are even more restrictive [7]. They have also indicated that a further lowering to 5TE% may be warranted, as currently recommended by the 2015 Scientific Advisory Committee on Nutrition (SACN) [8]. Although the adverse effects of sugars from SSB are well-established, there is a dearth of literature on associations of sugars from solid sources and health outcomes. Furthermore, robust evidence to support a defined limit of free sugar intake from solid foods is currently lacking.

An overview of recent meta-analyses [15] demonstrated positive associations of T2DM with higher consumption of SSB but lower intake of some sugar-sweetened foods. In children, an analysis of National Health and Nutrition Examination Survey (NHANES) data demonstrated SSB to be positively associated with body mass index (BMI); however, sugars from foods were inversely associated with BMI [12]. In older adults, an analysis of two Swedish cohorts demonstrated mortality to be inversely associated with (solid) ‘treats’ but positively associated with SSB [13].

Few studies have examined the association between free sugars from solid sources and health outcomes. The aforementioned studies did not examine thresholds of free sugar intake, as often referred to in the existing guidelines, but rather studied associations with the frequency of consumption for specific solid foods and beverages rich in free sugars. Moreover, no studies, to our knowledge, have examined the association between free sugars from solid sources and either overweight or the onset of diabetes during pregnancy. We examined associations of free sugars above various defined thresholds compared to below these thresholds, both with overweight at baseline and with GDM as occurred over a 13-year follow-up period in those who delivered; we assessed thresholds separately for solid and liquid sources.

We focused on a cohort of young to middle-aged girls and women who were at or would reach reproductive age at some point during the follow-up period. Reproductive age, according to the WHO, ranges from 15 to 50 years of age [16]. We included those 12 to 50 years at baseline. Overweight and GDM are two important and interrelated outcomes in female youth and young to middle-aged women; both conditions are indicators of risk for future cardiometabolic disease [17–21]. In this young population, long-term outcomes such as cardiovascular disease and related mortality, are rare [21]. In contrast, the prevalence of overweight ranges from ~ 25–30% among women in Canada [22, 23]. GDM is a condition that complicates up to 3–5% of pregnancies in Canada [24] and 10% of all pregnancies worldwide [25], and can be captured via in-patient discharge diagnostic codes [26]. We leveraged the linkage of 2004–2005 Canadian Community Health Survey (CCHS) 2.2 data, which included a 24-h dietary recall, with the Canadian hospital Discharge Abstract Database (DAD, 2004–2017), which included ICD-10 diagnostic codes, to conduct the present study.

Given the 5TE% and 10TE% recommended limits for intake of free sugars in existing guidelines [5–8], we examined associations of intakes of free sugars from solid and from liquid sources above compared to below these thresholds. We additionally evaluated a 2.5TE% threshold, given that we are stratifying for free sugars based on their source (solid foods vs. beverages) rather than overall intake. We further explored a 15TE% threshold.

## Methods

The study cohort included female participants (12–50 years old) without diabetes from Cycle 2.2 of the 2004–2005 CCHS who agreed to data linkage with the 2004–2017 DAD. The entire study cohort was used in cross-sectional analyses examining associations with overweight at baseline. Those with a delivery during the follow-up period were included in nested case-control analyses evaluating associations with GDM during the follow-up period.

### Ethics

The CCHS was approved by the Health Canada Research Ethics Board. Prior to data collection, Statistics Canada obtained written informed consent from all participants and separate consent for linkage to DAD. The Social Sciences and Humanities Research Council of Canada approved the present analyses (17-SSH-MCG-5265). We performed all analyses at the McGill-Concordia Quebec Inter-University Center for Social Statistics (QICSS).

### Data sources and variable definitions

The CCHS is a nation-wide, biennial survey with a multistage stratified cluster design [27]. In addition to the recurring general questionnaire on health determinants and healthcare utilization, Cycle 2.2 incorporated a 24-h dietary recall and direct measurements of height and weight. In-person, computer-assisted interviews were performed between January 14, 2004 and January 21, 2005. Statistics Canada grouped foods and beverages by Bureau of Nutritional Sciences categories, applying information from the Canadian Nutrient File to estimate TE, total sugars, macronutrients and other dietary components. We applied Bernstein and colleagues’ item-specific estimates of % total sugars that are free sugars in foods and beverages available in Canada [28]. We summed intake of free sugars (grams) for each participant, separately for solids and liquids. Liquids included beverages and the following fluid items: sauces, honey, syrups, gravies, soups, and creams. We multiplied grams/day by 4 kcal/gram and then divided by the total 24-h energy intake to express free sugars as a percent of TE. We variously stratified intakes of free sugars from solid and liquid sources across 2.5, 5, 10, and 15TE% thresholds. In a secondary analysis, we classified free sugars, respective of their source, into 4 mutually exclusive categories (<5TE%, 5 to 10TE%, 10 to 15TE%, >15TE%). As part of CCHS procedures, a random sample of respondents were also randomly selected to complete a second 24-h dietary recall within 3–10 days after the initial interview [27]. In this subgroup, we compared the proportion of participants categorized above each free sugar threshold across the two recalls to support the use of a single 24-h recall as a robust measure of intake.

We defined overweight in adolescents (12–19 years old) as BMI ≥ 85th percentile (sex and age-specific [29]) and in adults (20 years and older) as BMI ≥25 kg/m^2^. When direct measures of height and/or weight were unavailable, we included self-reported height and/or weight to calculate BMI with application of a correction factor (BMI_measured_ = − 0.12 + 1.05*BMI_self-reported_) [30]. This correction factor was developed using anthropometric data from the 2005 CCHS, in order to adjust self-reported estimates of BMI to more closely approximate measured values. Application of this correction factor improved the sensitivity among women classified in the overweight category, based on self-reported data, from 62.6 to 79.7% in another study [30].

Dietary covariates included fats (saturated, monounsaturated and polyunsaturated; expressed as TE%), protein (TE%), sodium (grams), potassium (grams), fibre (grams), non-sugar carbohydrates (TE%) and TE intake (kcal) were extracted from 24-h dietary recalls. We also accounted for daily intake of 5 or more servings of fruit and vegetables and daily intake fruit juice, derived from the general questionnaire. Other variables (derived from the CCHS 2.2 survey [27]) considered were age, ethnicity (dichotomized as Caucasian or non-Caucasian based on self-identified background), immigrant status (immigrant or non-immigrant), food insecurity (moderately or severely food insecure vs. food secure), urban-rural residence, physical activity (physical activity index collapsed as active vs. inactive if < 1.5 metabolic equivalents/kg/day based on self-reported exercise type, duration and frequency), smoking status (current/non-smoker) and self-characterization of food intake reported compared to usual intake (much more, typical, much less). These have previously been associated with sugar intakes and/or overweight [31–35].

Statistics Canada linked 95% of CCHS responses to DAD data in individuals who provided consent for data sharing (83%) with provincial Ministries of Health and Health Canada [36, 37]. DAD includes hospital discharge diagnoses (International Classification of Diseases 10th revision codes [ICD-10]) from all Canadian provinces except Quebec.

### Participants and case ascertainment

Our focus was female participants who were of reproductive age or who would reach reproductive age during the thirteen-year follow-up period. Therefore, we excluded boys and men, women older than 50 years, and girls less than 12 years at baseline. We also excluded those without dietary recall information and/or anthropometric measures (i.e., neither measured nor self-reported), those with diabetes, and those who were pregnant/breastfeeding at assessment. The remaining participants were included in the analyses examining associations between free sugars and overweight at baseline.

Within this cohort, we delineated a ‘delivery cohort,’ identifying participants with an ICD-10 code for an in-hospital delivery (Supplemental Table 1) between March 30, 2004 and December 14, 2017, in order to examine associations between free sugars and GDM status. We excluded those with codes for diabetes that developed sometime between baseline evaluation and pregnancy (i.e., computing back 9 months from delivery date to ascertain pregnancy period). We classified cohort members as having (cases) or not having (controls) a diagnosis of GDM recorded at discharge following delivery. In Canada, GDM-specific inpatient codes have demonstrated 86% sensitivity and 99% specificity [38].

### Statistics

We calculated descriptive statistics stratified by weight status at baseline and stratfied by GDM status in the delivery cohort. As previously noted, current guidelines [5–8] variously recommend limiting added or free sugars to 5TE% or 10TE%. Therefore, in a series of multivariable logistic regression models, we separately evaluated associations of free sugars from solids and from liquids with overweight at baseline across 5TE% and 10TE%, and additionally opted to evaluate 2.5TE% and 15TE% thresholds. We evaluated associations with GDM during the follow-up period in case-control analyses across the 2.5TE%, 5TE% and 10TE% thresholds; associations between GDM and the 15TE% threshold of free sugar intake (stratified by source) were not examined due to the low number of participants who delivered and consumed above this threshold. Odds ratios (OR) with 95% CI were computed.

Models were adjusted for age, ethnocultural background, immigrant status, food insecurity, urban-rural residence, physical activity, smoking, thresholds of %TE from the alternate source (i.e., models assessing free sugars from solid sources at the 5TE% threshold were adjusted for free sugars from liquids at an identical TE% threshold), self-reported food intake compared to usual intake and dietary covariates described above. Associations of free sugars with GDM status were additionally adjusted for overweight in a secondary analysis. McNemar’s test (*p*-value statistic) was conducted to assess the agreement of free sugar categorization (proportion of participants categorized above each free sugar threshold) from the first 24-h recall interview among those with a second 24-h recall interview. In another secondary analysis, we classified free sugars into 4 mutually exclusive categories (<5TE%, 5 to 10TE%, 10 to 15TE%, >15TE%), and examined for dose-response associations with both overweight and with GDM; individuals consuming free sugars <5TE% served as the reference group. Lastly, we conducted a sensitivity analysis which examined associations between solid and liquids sources of free sugars with overweight and with GDM case status without adjustment for TE intake.

Analyses were conducted at the McGill University site of the Canadian Research Data Centre Network using SAS version 9.3 (SAS Institute, Inc., Cary, North Carolina). Consistent with the policy to protect confidentiality of participants, tabulations with cell counts under 30 individuals were not released [39].

## Results

**Fig. 1:**
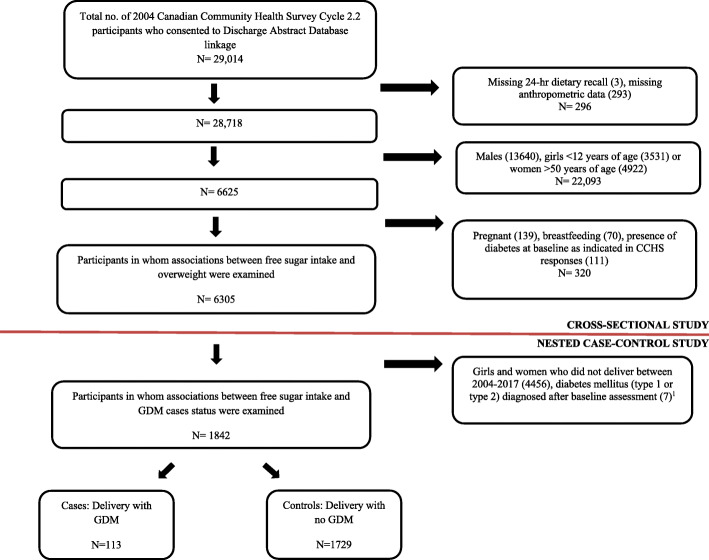
Participant flow diagram. ^1^We excluded cases of diabetes mellitus (type 1 or type 2) that were diagnosed at the time of hospital discharge but developed sometime between baseline assessment and pregnancy (9 months prior to delivery) during the follow up period (March 30, 2004 – December 14, 2017)

Following exclusions, there were 6305 participants (Fig. 1) among whom 1842 delivered and did not develop diabetes between baseline assessment and pregnancy. Approximately three quarters (71.6%) of participants had direct measures of weight and height at baseline. The mean interval between baseline assessment and delivery was 7.6 years (standard deviation, SD, 3.7 years).

There were 2226 participants (35%) of the study population who had records of a second 24-h recall. McNemar’s test generated *p*-values > 0.1 across all thresholds of free sugar intake (Supplemental Table 2), demonstrating that differences in the proportion of participants categorized above each respective free sugar threshold were not statistically significant across both dietary recalls.

Forty percent (*n* = 2505) of participants were overweight (Table 1). They were, on average, older and included a lower proportion of immigrants than those who were not overweight. A higher proportion were food insecure. A greater proportion smoked cigarettes and were physically inactive. A lower proportion consumed fruit juice and/or 5 or more servings of fruits and vegetables daily. Free sugars accounted for 18TE% on average, with ~8TE% consumed from solid sources and ~ 10TE% from liquid sources. In addition, participants who were overweight demonstrated similar intakes of free sugars derived from solid and/or liquid sources compared to those who were not overweight. In approximately half of the study participants, free sugars from solid sources (57.1%) and from liquid sources (56.7%) was above 5TE% (Supplemental Fig. 1).

**Table 1:** Sociodemographic and dietary characteristics at baseline, stratified by overweight at baseline and by GDM status during the follow-up period

Baseline characteristics	Stratified by weight status at baseline (*N* = 6305)		Delivery between 2004 and 2017 (*N* = 1842)	
	Overweight ***N*** = 2505	Not overweight N = 3800	Cases (GDM) ***N*** = 113	Controls (no GDM) ***N*** = 1729
Overweight, number (%)^1^	2505 (100)	0 (0)	63 (55.6)	568 (32^.^9)
Age, years, mean (SD)	30.3 (1.24)	21.1 (2.30)	22.8 (7.16)	20.6 (5.70)
Caucasian, number (%)^2^	2177 (86.9)	3279 (86.3)	85 (75.2)	1475 (85.3)
Immigrant, number (%)	155 (6.19)	364 (9.58)	13 (11.5)	113 (6.53)
Food insecure, number (%)	322 (12.9)	274 (7.21)	17 (15.0)	119 (6.88)
Rural residence, number (%)	590 (23.6)	781 (20.5)	19 (16.8)	370 (21.4)
Current smoker, number (%)	746 (29.8)	823 (21.7)	34 (30.1)	446 (25.6)
Active, number (%)	1117 (44.6)	2103 (55.3)	56 (49.6)	926 (53.6)
BMI, kg/m^2^, mean (SD)	30.5 (5.90)	21.2 (2.31)	27.1 (6.78)	24.5 (5.73)
Total energy intake kcal, mean (SD)	1815 (793)	1996 (850)	1885 (868)	1940 (849)
Amount of food intake reported in the last 24 h compared to usual intake, number (%)^3^	Much more: 197 (7.86)	Much more: 244 (6.42)	Much more: 9 (7.96)	Much more: 149 (8.62)
	Typical: 1821 (72.7)	Typical: 2967 (78.0)	Typical: 88 (77.9)	Typical: 1307 (75.6)
	Much less: 484 (19.3)	Much less: 588 (15.5)	Much less: 16 (14.2)	Much less: 273 (15.8)
Free Sugar Intake: % of total energy, mean (SD)				
Overall free sugars	17.2 (12.9)	18.1 (12.9)	14.8 (12.1)	18.5 (13.6)
Free sugars in solids	7.90 (7.80)	8.30 (7.60)	5.60 (5.40)	7.81 (7.9)
Free sugars in liquids	10.2 (11.0)	10.5 (10.9)	10.2 (11.5)	11.6 (11.9)
Non-sugar carbohydrates^4^	27.9 (9.20)	28.2 (9.90)	28.3 (10.3)	28.4 (10.2)
Fats and Proteins: % of total energy, mean (SD)				
Saturated fats	10.2 (4.25)	10.4 (4.25)	11.3 (6.55)	10.2 (4.20)
Monounsaturated fats	12.5 (4.75)	12.2 (4.47)	13.1 (5.05)	12.3 (4.62)
Polyunsaturated fats	5.54 (2.76)	5.40 (2.68)	5.64 (2.73)	5.37 (2.75)
Protein	15.7 (6.16)	14.9 (5.41)	16.0 (6.94)	14.8 (5.49)
Other, mean (SD)				
Sodium, g	2.72 (1.47)	2.91 (1.61)	3.68 (2.34)	3.79 (2.30)
Potassium, g	2.60 (1.24)	2.77 (1.34)	3.27 (1.79)	3.44 (1.97)
Fibre, g	13.7 (8.30)	14.9 (8.90)	16.8 (10.3)	18.2 (11.7)
Daily fruit juice, number (%)	1055 (42.1)	2063 (54.3)	57 (50.4)	859 (49.7)
≥ 5 servings of fruit and vegetables daily, number (%)	660 (26.3)	1193 (31.4)	22 (19.5)	433 (25.0)

^1^Overweight in adolescents (12–19 years old) was defined as corresponding to BMI above the 85th percentile. BMI percentiles were age- and sex- standardized in accordance with Center for Disease Control and Prevention (CDC).^18^ Adults (20 years and older) were classified as overweight at a BMI equal to or above 25 kg/m^2^.

^2^Ethnicity was based on self-identified ethnocultural group. Among non-Caucasians, South Asians, Chinese, Blacks and Latin Americans comprised 27, 23, 19 and 6%, respectively.

^3^Free sugars are defined as all monosaccharides and disaccharides added to foods by the manufacturer, cook or consumer, plus sugars naturally present in honey, syrups and fruit juices. Liquid sources of free sugars from the CCHS survey included beverages, bottled sauces, honey and syrups, gravies, soups, and creams.

^3^Responses for this variable were self-reported by participants when completing the 24-h dietary recall interview. 4 participants reported “do not know” in their response (overweight: 3, not overweight: 1).

^4^Non-sugar carbohydrates were defined as total carbohydrate intake – total sugar intake – total fibre intake and expressed as percent of total energy intake.

Among those who delivered, there were 113 with GDM (cases) and 1729 without GDM (controls). Compared to controls, the cases included a higher proportion who were immigrants and a lower proportion who were white. A higher proportion were food insecure. A greater proportion smoked cigarettes, were physically inactive, and consumed less than 5 or more servings of fruits and vegetables daily. Among the cases, free sugars from both solid and liquid sources, were, on average, slightly less than intake from the controls (Table 1). Overall, a higher proportion of participants consumed free sugars from liquid sources (65.5%) relative to solid sources (54.8%) above the 5TE% threshold (Supplemental Fig. 2).

The predominantly reported specific solid sources were white/brown sugar added to food during preparation, canned fruit, processed cheese, bread products and cereals (Supplemental Fig. 3). The primary liquid sources of free sugars were bottled sauces (i.e., ketchup), fruit juice, sweetened coffee and SSB (Supplemental Fig. 4). Sweetened tea and sweetened coffee categories were distinct from white/brown sugar, unsweetened tea, and unsweetened coffee.

### Associations of overweight with free sugars

Free sugars from solid sources were associated with slightly lower odds of overweight above vs. below the 2.5TE% threshold (OR 0.80, 95% CI 0.70–0.92), above vs. below the 5TE% threshold (OR 0.89, 95% CI 0.79–0.99) and above vs. below the 10TE% threshold (OR 0.86, 95% CI 0.75–0.97). Free sugars from liquid sources were associated with higher odds of overweight above vs. below the 2.5TE% threshold (OR 1.20, 95% CI 1.07–1.36), above vs. below the 10TE% threshold (OR 1.17, 95% CI 1.02–1.34), and above vs. below the 15TE% (OR 1.43, 95% CI 1.23–1.67) threshold (Table 2). In a secondary analysis that grouped free sugar intakes into four mutually exclusive categories, consumption of free sugars >15TE% from liquid sources was associated with increased odds of overweight (OR 1.39, 95% CI 1.17–1.66; Supplemental Table 3) relative to those consuming <5TE%. Findings from our sensitivity analysis (Supplemental Table 4), which did not adjust for TE intake, demonstrated similar estimates with the primary analysis examining associations with overweight (Table 2).

**Table 2:** Baseline associations between overweight and free sugars, from solid and liquid sources

	Associations with overweight at baseline (*N* = 6305)^a^			
	No. (%) Overweight^b^ (***N*** = 2505)	No. (%) Not overweight (***N*** = 3800)	Unadjusted OR (95% CI)	Adjusted OR (95% CI)^**3**^
SOLID SOURCES OF FREE SUGARS^a^				
> 2.5TE%	1879 (75)	3040 (80)	0.75 (0.67–0.85)	0.80 (0.70–0.92)
< 2.5TE%	626 (25)	760 (20)		
> 5TE%	1363 (54)	2243 (60)	0.83 (0.75–0.92)	0.89 (0.79–0.99)
< 5TE%	1142 (46)	1557 (41)		
> 10TE%	664 (27)	1157 (30)	0.82 (0.74–0.92)	0.86 (0.75–0.97)
< 10TE%	1841 (73)	2643 (70)		
> 15TE%	356 (14)	549 (14)	0.98 (0.85–1.13)	1.10 (0.93–1.31)
< 15TE%	2149 (86)	3251 (86)		
LIQUID SOURCES OF FREE SUGARS^a^				
> 2.5TE%	1578 (63)	2546 (67)	0.84 (0.75–0.93)	1.20 (1.07–1.36)
< 2.5TE%	927 (37)	1254 (33)		
> 5TE%	1343 (54)	2232 (59)	0.81 (0.73–0.90)	1.09 (0.96–1.25)
< 5TE%	1162 (46)	1568 (41)		
> 10TE%	939 (37)	1500 (39)	0.92 (0.83–1.02)	1.17 (1.02–1.34)
< 10TE%	1566 (63)	2300 (61)		
> 15TE%	622 (25)	909 (24)	1.05 (0.93–1.18)	1.43 (1.23–1.67)
< 15TE%	1883 (75)	2891 (76)		

^a^Each threshold of free sugar intake (2.5TE%, 5TE%, 10TE%, 15TE%) was included in a separate regression model. We compared those with intake above each of these set thresholds to individuals consuming below each respective threshold (reference group). Regression models were adjusted for age, ethnicity, immigrant status, food insecurity, rural residence, smoking, physical activity, total energy intake, amount of food intake reported in the last 24 h compared to usual intake, consumption of fruit juice daily and ≥ 5 servings of fruit and vegetables and other dietary covariates (e.g., fats, protein, sodium, potassium, fibre and non-sugar carbohydrates)

^b^Overweight in adolescents (12–19 years old) was defined as corresponding to BMI above the 85th percentile. BMI percentiles were age- and sex- standardized in accordance with Center for Disease Control and Prevention (CDC). Adults (20 years and older) were classified as overweight at a BMI equal to or above 25 kg/m^2^

### Associations of GDM with free sugars

Those with intakes of free sugars from solid sources above 5TE% had 44% less likely odds to develop GDM (OR 0.56, 95% CI 0.36–0.85; Table 3) relative to participants with consumption below this threshold. In a secondary analysis, those who consumed 5 to 10TE% as free sugars from solid sources had 42% less likely odds to develop GDM compared to those whose consumption was below 5TE% (OR 0.58, 95% CI 0.35–0.95; Supplemental Table 3). No conclusive associations between GDM and free sugars from liquids were observed (Table 3). Being overweight was associated with a more than two-fold increase for GDM (OR 2.58, 95% CI 1.71–3.90; Supplemental Table 5). Findings from the sensitivity analysis (Supplemental Table 4), which did not adjust for TE intake, demonstrated increased point estimates but the overall trends are similar to the primary analysis examining associations with GDM (Table 3).

**Table 3:** Crude and multivariable associations between free sugars (stratified by solid and liquid sources) and GDM

	Delivery between 2004 and 2017				
	Associations with GDM case status during follow-up (***N*** = 1842)^a^				
	No. (%) of Cases (***N*** = 113)	No. (%) of Controls (***N*** = 1729)	Unadjusted OR (95% CI)	Adjusted OR (excluding ‘overweight’ from multivariable model)	Adjusted OR (including ‘overweight’ in multivariable model)^b^
SOLID SOURCES OF FREE SUGARS^a^					
> 2.5TE%	80 (71)	1300 (75)	0.80 (0.53–1.22)	1.05 (0.64–1.71)	1.08 (0.67–1.73)
< 2.5TE%	33 (29)	429 (25)			
> 5TE%	43 (38)	967 (56)	0.48 (0.33–0.72)	0.56 (0.36–0.85)	0.60 (0.39–0.92)
< 5TE%	70 (62)	762 (44)			
> 10TE%	23 (30)	449 (26)	0.73 (0.46–1.17)	0.73 (0.42–1.28)	0.79 (0.45–1.40)
< 10TE%	90 (80)	1280 (74)			
LIQUID SOURCES OF FREE SUGARS^a^					
> 2.5TE%	84 (74)	1323 (77)	0.89 (0.57–1.38)	0.92 (0.62–1.45)	0.94 (0.61–1.47)
< 2.5TE%	29 (26)	406 (23)			
> 5TE%	65 (58)	1141 (66)	0.70 (0.47–1.03)	0.89 (0.57–1.38)	0.91 (0.58–1.42)
< 5TE%	48 (42)	588 (44)			
> 10TE%	53 (47)	831 (48)	0.95 (0.65–1.40)	1.06 (0.67–1.69)	1.07 (0.66–1.71)
< 10TE%	60 (53)	898 (52)			

^a^Each threshold of free sugar intake (2.5TE%, 5TE%, 10TE%) was included in a separate regression model. Associations between GDM and free sugar intake at the 15TE% threshold were not examined due to inadequate statistical power at this level of intake. We compared those with intake above each of these set thresholds to individuals consuming below each respective threshold (reference group). Regression models were adjusted for age, ethnicity, immigrant status, food insecurity, rural residence, smoking, physical activity, total energy intake, amount of food intake reported in the last 24 h compared to usual intake, consumption of fruit juice daily and ≥ 5 servings of fruit and vegetables and other dietary covariates (e.g., fats, protein, sodium, potassium, fibre and non-sugar carbohydrates)

^b^Overweight in adolescents (12–19 years old) was defined as corresponding to BMI above the 85th percentile. BMI percentiles were age- and sex- standardized in accordance with Center for Disease Control and Prevention (CDC). Adults (20 years and older) were classified as overweight at a BMI equal to or above 25 kg/m^2^

## Discussion

In a large cohort of girls and young to middle-aged women, free sugars from liquid sources were associated with overweight. We observed a consistent, positive association across all thresholds examined. In contrast, free sugars from solid sources were associated with lower odds of overweight across the 2.5TE%, 5TE% and 10TE% thresholds. Approximately one third of the cohort delivered during the 13-year follow-up period, including 6% (113 participants) with GDM. With respect to free sugars, the only association identified with GDM was lower odds of GDM among those consuming more than 5TE% as free sugars from solid sources, compared to those consuming below this threshold. This was consistent with a secondary analysis in which free sugar intake from solid sources at 5 to 10TE% was associated with lower odds of GDM compared to intake less than 5%. Our findings do not support specific guidelines to limit intakes of free sugars from food sources but do support the limitation of intake from liquid sources in girls and women 12 to 50 years of age.

In these girls and women, in line with the evidence from guidelines for free sugar consumption, which are based on evidence for SSB [5, 8, 40], we observed a consistent increase in the risk of overweight across all thresholds of free sugar intake from liquid sources (Table 2). Overweight is an established risk factor for a variety of adverse outcomes, including GDM [1], type 2 diabetes [1], hypertension [41], cardiac disease [42], stroke [42], various forms of cancer [43], osteoarthritis [44], and depression [45]. Although we were not powered to demonstrate a specific association of free sugars from liquid sources with GDM, we identified both an association of free sugars from liquid sources with overweight and a strong association of overweight with GDM. In contrast, we did not demarcate a threshold of harm for free sugars from solid sources in this younger group of women. Instead, we identified what might be termed a ‘sweet spot’ for free sugars from solids as being somewhere between 5-10TE%, a level that signalled inverse associations with overweight and GDM, compared to lower levels of consumption (Supplemental Table 3). It is possible that at least in this age group of girls and women, such an intake of free sugars from solid sources is simply the amount stemming from consuming a reasonably healthy diet with a moderate sugar content. However, the mechanisms underlying the inverse associations among women of reproductive age requires further investigation.

Our findings in girls and young to middle-aged women are consistent with an emerging body of literature signaling adverse effects of free sugars from liquid sources, but an absence of such a signal for free sugars from solid sources, at least among the limited studies that have evaluated related issues. Moreover, these studies have examined different demographic groups than included in our study [12, 13, 15]. An overview of meta-analyses [15] examining relationships between specific sources of sugars and T2DM in adults reported inverse associations between several sugar-containing food items and T2DM, while SSB were positively associated with T2DM. A cross-sectional analysis of 2009–2014 NHANES data in children [12] reported higher free sugars from solid sources to be inversely associated with BMI z-score (− 0.03 increment [95% CI: − 0.04 to − 0.02] per TE% increase), whereas free sugars from SSB were positively associated with BMI (0.01 increment [95% CI: 0.002 to 0.03] per TE% increase). A prospective analysis of two Swedish cohorts [13] ascertained a 17% lower mortality (HR 0.83, 95% CI 0.74–0.93) in the highest vs. lowest categories of ‘treat’ consumption (i.e., sugar-containing foods), but a 14% higher mortality (HR 1.14, 95% CI 1.03–1.26) in the highest vs. lowest SSB consumption categories. Taken as a whole, these studies, including ours, indicate that specific limitations on consumption of free sugars from solid sources may not be necessary, at least in certain age groups or particular areas of the world.

The reasons for differences in associations for solid versus liquid sources of free sugars are not clear. One possibility is differences in effects of sucrose and fructose. Foods are generally sweetened with sucrose (50% glucose: 50% fructose) while high-fructose corn syrup (45% glucose: 55% fructose; contains 10% more fructose) is commonly used in SSB [46]. Fructose is primarily metabolized in the liver and large amounts may lead to postprandial hypertriglyceridemia, resulting in visceral adiposity and insulin resistance [47]. In one meta-analysis [48], CVD mortality was inversely associated with sucrose but positively associated with fructose. The authors noted that much of the sucrose in diets tend to be from healthy solid food sources that are rich in fibre and nutrients (e.g., grain products), [48], consistent with our observations (Supplemental Fig. 3).

To date, guidelines that support limiting dietary free sugars are based on evidence for SSB, not on evidence related to solid (food) sources of free sugars (i.e., DGAC, SACN) [5, 8, 40]. No current guidelines distinguish between specific levels of free sugars derived from solid and liquid sources. The DGAC advocates limiting added sugars to 10TE% [5]. The WHO suggests a 10% limit for free sugars [7] while the SACN guidelines recommend a 5TE% limit [8]. We determined no threshold of harm for free sugars from solids; intakes above 2.5TE%, 5TE% and 10TE% thresholds were associated with lower overweight risk while intakes above 5TE% were associated with lower GDM risk, relative to intake below each of these thresholds, consistent with other studies discussed that considered different but related outcomes. Thus, a growing body of literature [47, 49] suggests that with respect to free sugars, we should focus guidelines on SSB rather than specify restrictions on sugars from food sources, given the current absence of evidence for harm.

Our study has some limitations. Although we adjusted for TE, its measurement is prone to error [27]. A previous crossover trial demonstrated higher intake following liquid sugar ‘preloads’ than solid ones of identical energy content [50]. In our study, a higher than computed TE in those consuming free sugars from liquid sources could have contributed to the positive association between free sugars from liquids and overweight observed, given weaker satiety effects of liquids reported by several investigators compared to solids [10, 15, 50–53]. Nonetheless, this would not explain the *inverse* associations with free sugars from solids that we and others have observed.

We also relied on a single, 24-h dietary recall at baseline to derive free sugar intake; however, among participants who had records of a second 24-h recall, we observed that McNemar’s test generated *p*-values > 0.10. These findings support the null hypothesis that the proportion of individuals categorized above each free sugar thresholds across both dietary recalls are in agreement and that the difference was not statistically significant.

We conducted two separate types of models based on free sugar intake from liquid and solid sources, above vs. below various thresholds, and, in a secondary analysis, separated into four mutually exclusive categories, with <5TE% as the reference group. The latter analyses resulted in smaller numbers within each category and thus less power to detect associations, compared to the primary analyses in which we dichotomized intake as above vs. below several different thresholds in separate models. Nonetheless, these findings suggest a dose-response pattern for adverse associations of free sugars from liquid sources with overweight, but are conclusive only for the >15TE%. It was also notable that not only was consumption of free sugars from solids above vs. below a 5TE% threshold inversely associated with GDM but also that consumption of between 5 and 10 TE% was associated with lower GDM odds than consumption under 5TE%.

In our study, 7.6 years lapsed between baseline assessment and delivery. While it is possible for intake to change over time, previous longitudinal, nutritional-tracking studies have demonstrated that diets tend to remain relatively stable, even over the transitioning years from childhood to adulthood [54]. Another potential limitation is reporting bias; for example, overweight individuals may underreport intake. To offset this, we adjusted for TE, an approach adopted by others [13, 27]. In addition, we performed sensitivity analyses that did not adjust for TE intake in models examining associations with both overweight and GDM (Supplemental Table 4), in order to assess the robustness of our adjusted effect estimates. Findings from our sensitivity analyses were similar to the estimates resulting from our primary analyses. It is possible that the inverse associations between overweight and free sugars from solids that we identified may have resulted from reverse causation, with overweight individuals consuming less sugar-containing food due to concerns about their weight. However, our detection of consistent, positive associations between free sugars from liquids and overweight suggest that underreporting was not a major issue.

Finally, we acknowledge that our study includes girls and women of reproductive age across a broad range (12–50 years). With the rising prevalence of both overweight and GDM among young women [22, 23], it is critical to study these early, powerful indicators of risk for future cardiometabolic disease [17–21]. As noted previously, we sought to include young to middle-aged participants who could become pregnant at any time point during the 13-year follow-up period in order to study associations with both overweight and GDM. Approximately 10% of the overall cohort was 46 to 50 years of age at baseline. For the sub-cohort of women who delivered, 5% of women were 35 years of age or older (i.e., more than 90 women) and 1% were at 49 to 50 years (i.e., ~ 20 women); retaining these women allowed us to maximize our power to detect associations of free sugars with overweight and GDM. Caution is needed in interpreting the results as the implications of our study are limited to this population.

A larger cohort of women who delivered could potentially elucidate conclusive associations with GDM beyond the 10TE% threshold. Our findings with regards to free sugars from solids sources remain inconclusive at this threshold likely due to low numbers of GDM cases consuming free sugars specifically from solid food sources above this level. However, our analyses were sufficiently powered to demonstrate conclusive findings of lower odds of both overweight and GDM with intakes above vs. below a 5TE% threshold for free sugars from solid sources. This is likely due to the distribution of free sugar intake from solid sources in the study cohort (Supplemental Figs. 1 and 2), which allow for a well-balanced comparison among participants consuming above or below this limit. Strengths of our study include the use of 24-h dietary recall by trained interviewers linked with health outcome data using validated health administrative database definitions and a large nationally representative sample of women with follow-up to 13 years. In addition to dietary data, the CCHS included direct measures of height and weight as well as several key demographic variables.

In summary, our results do support limitation of free sugars from liquid sources. We did not demarcate a threshold of harm for free sugars from solid sources. Considered with emerging findings from several other studies in younger and older populations, there remains insufficient evidence to recommend specific guidelines that restrict intakes of free sugars from solid sources. In contrast, adverse effects of sugars from SSB are consistent across the literature. Given that our study, to our knowledge, is the first to examine differential source-specific effects of free sugar on the onset of GDM, any potential benefits of foods containing free sugars need to be confirmed in this population. Our findings address a key knowledge gap in the literature will help inform policymakers and contributes to a growing body of evidence that raises questions around the assumption that free sugars, irrespective of source, are harmful.

## Supplementary Information

**Additional file 1: Supplemental Table 1**. ICD-10 Diagnostic Codes Used to Identify Outcomes from the National 2004–2017 Discharge Abstract Database. **Supplemental Table 2**. Comparison between participants who had completed both 1st and 2nd 24-h dietary recalls. **Supplemental Table 3**. Secondary analysis: Multivariate associations between solid and liquid sources of free sugars with overweight and with GDM case status when adjusting for mutually exclusive categories of free sugar intake. **Supplemental Table 4**. Sensitivity analysis: Multivariate associations between solid and liquids sources of free sugars with overweight and with GDM case status without adjustment for total energy intake*.* **Supplemental Table 5**. Multivariate associations between baseline characteristics with overweight and with GDM case status. **Supplemental Figure 1**. Percent of all participants above various thresholds of free sugar (FS) intake as a percent of total energy (TE%)^1^. **Supplemental Figure 2**. Percent of delivery cohort above various thresholds of free sugar (FS) intake as a percent of total energy (TE%)^1^. **Supplemental Figure 3**. Top sources of free sugars from solid sources. **Supplemental Figure 4**. Top sources of free sugars from liquid sources.

## Data Availability

The data made available for linkage are only accessible through the Canadian Research Data Centre Networks with permission from Statistics Canada. The data that support the findings of this study are not available from the authors as they do not hold the data files. All analyses were conducted by the authors at a Statistics Canada Research Data Centre, a secure physical environment available to accredited researchers in Canada for research purposes. These centres are located on university campuses across Canada and are staffed by Statistics Canada employees. For further information, please see https://www.statcan.gc.ca/eng/microdata

## Abbreviations

BMI: Body Mass Index
CCHS: Canadian Community Health Survey
DGAC: Dietary Guidelines Advisory Committee
DAD: Discharge Abstract Database
FS: Free sugars
GDM: Gestational diabetes mellitus
NHANES: National Health and Nutrition Examination Survey
QICSS: Quebec Inter-University Center for Social Statistics
SACN: Scientific Advisory Committee on Nutrition
SSB: Sugar-sweetened beverages
TE: Total energy
TE%: Percent of total energy
T2DM: Type 2 diabetes mellitus
