# Supplementary material for: Associations of overweight and gestational diabetes mellitus with free sugars from solid and liquid sources: cross-sectional and nested case-control analyses
Source: BMC Public Health. 2021 Oct 23;21:1923. doi: 10.1186/s12889-021-12000-3 (PMC8539824; doi:10.1186/s12889-021-12000-3)
Supplement: Supplementary file 1 — Additional file 1: Supplemental Table 1. ICD-10 Diagnostic Codes Used to Identify Outcomes from the National 2004–2017 Discharge Abstract Database. Supplemental Table 2. Comparison between participants who had completed both 1st and 2nd 24-h dietary recalls. Supplemental Table 3. Secondary analysis: Multivariate associations between solid and liquid sources of free sugars with overweight and with GDM case status when adjusting for mutually exclusive categories of free sugar intake. Supplemental Table 4. Sensitivity analysis: Multivariate associations between solid and liquids sources of free sugars with overweight and with GDM case status without adjustment for total energy intake. Supplemental Table 5. Multivariate associations between baseline characteristics with overweight and with GDM case status. Supplemental Figure 1. Percent of all participants above various thresholds of free sugar (FS) intake as a percent of total energy (TE%)1. Supplemental Figure 2. Percent of delivery cohort above various thresholds of free sugar (FS) intake as a percent of total energy (TE%)1. Supplemental Figure 3. Top sources of free sugars from solid sources. Supplemental Figure 4. Top sources of free sugars from liquid sources. [file 12889_2021_12000_MOESM1_ESM.docx]

**SUPPLEMENTAL TABLE 1** ICD-10 Diagnostic Codes Used to Identify Outcomes from the National 2004-2017 Discharge Abstract Database

| **Outcome** | **ICD-10 codes** |
| --- | --- |
| In-hospital delivery | Z34-Z35, Z37-Z38, O10-16, O20-O48, O60-O75, O85-O92, O94, O98 to O99 |
| Diabetes | E10-E14, O24.5-O24.6 |
| Gestational diabetes mellitus | O24.8 |

**SUPPLEMENTAL TABLE 2** Comparison between participants who had completed both 1^st^ and 2^nd^ 24-hour dietary recalls

| N=2226 | 1^ST^ 24-hour dietary recall^1^ | 2^ND^ 24-hour dietary recall | McNemar’s p-value^2^ |
| --- | --- | --- | --- |
| >5TE% free sugars from solids, n (%) | 1239 | 1182 | 0.21 |
| >10TE% free sugars from solids, n (%) | 630 | 580 | 0.18 |
| >15TE% free sugars from solids, n (%) | 326 | 283 | 0.28 |
| >5TE% free sugars from liquids, n (%) | 1277 | 1244 | 0.31 |
| >10TE% free sugars from liquids, n (%) | 855 | 842 | 0.29 |
| >15TE% free sugars from liquids, n (%) | 547 | 516 | 0.10 |

^1^ The mean difference in free sugar intake from solid sources and liquid sources was 0.97TE% (95% CI 0.57-1.41) and 0.46TE% (-0.12-1.04), respectively, between 1^st^ and 2^nd^ dietary recall.

^2^ We conducted McNemar’s test to determine whether differences in the proportion of participants categorized above each free sugar threshold are statistically significant between the 1^st^ and 2^nd^ 24-hour dietary recall records among participants. H_0_ = the proportion of participants categorized above the respective free sugar threshold are not significantly different across dietary recalls. p value > 0.05 is interpreted as insufficient evidence to reject the null hypothesis.

**SUPPLEMENTAL TABLE 3** Secondary analysis: Multivariate associations between solid and liquid sources of free sugars with overweight and with GDM case status when adjusting for mutually exclusive categories of free sugar intake

|  |  | | | Delivery between 2004-2017 | | |
| --- | --- | --- | --- | --- | --- | --- |
|  | **Associations with overweight at baseline (N=6305)** | | | **Associations with GDM case status during follow-up**^‡^ **(N=1842)** | | |
|  | **No. (%) of overweight participants**  **(N=2580)** | **No. (%) of non- overweight participants (N=3725)** | **Adjusted odds ratio (95% CI)** | **No. (%) of Cases (N=113)** | **No. (%) of Controls (N=1729)** | **Adjusted odds ratio (95% CI)^2^** |
| Free sugars from solid sources as a percent of total energy | | | | | | |
| <5TE%^1^ | 1187 (46) | 1484 (40) | 1.00 | 70 (62) | 760 (44) | 1.00 |
| 5 to 10TE% | 808 (31) | 1238 (33) | 0.90 (0.79 to 1.03) | 26 (23) | 574 (33) | 0.58 (0.35 to 0.95) |
| 10 to 15TE% | 341 (13) | 555 (15) | 0.90 (0.76 to 1.07) | 11 (10) | 227 (13) | 0.64 (0.32 to 1.28) |
| >15TE | 244 (10) | 448 (13) | 0.82 (0.67 to 1.00) | 6 (5) | 166 (10) | 0.54 (0.22 to 1.36) |
| Free sugars from liquid sources as a percent of total energy | | | | | | |
| <5TE%^1^ | 1412 (55) | 1922 (52) | 1.00 | 56 (50) | 814 (47) | 1.00 |
| 5 to 10TE% | 409 (16) | 761 (20) | 0.97 (0.84 to 1.13) | 18 (16) | 316 (18) | 0.97 (0.49 to 1.55) |
| 10 to 15TE% | 308 (12) | 489 (13) | 1.16 (0.93 to 1.33) | 19 (17) | 253 (15) | 1.11 (0.61 to 2.01) |
| >15TE | 428 (17) | 538 (14) | 1.39 (1.17 to 1.66) | 18 (16) | 330 (19) | 0.76 (0.40 to 1.47) |

^1^<5E% served as the reference group in the secondary analysis. All categories were included as predictors in the regression model.

^2^ Estimates presented from these secondary analyses were not adjusted for overweight given that this BMI may mediate the relationship between free sugar intake and GDM.

**SUPPLEMENTAL TABLE 4** Sensitivity analysis: Multivariate associations between solid and liquids sources of free sugars with overweight and with GDM case status without adjustment for total energy intake

|  | *Overweight* | GDM case status | |
| --- | --- | --- | --- |
|  | **Adjusted OR (95% CI) excluding ‘total energy intake’ from multivariable model** | **Adjusted OR (95% CI) excluding ‘total energy intake’ and ‘overweight’ from multivariable model** | **Adjusted OR (95% CI) excluding ‘total energy intake’ from multivariable model** |
| SOLID SOURCES OF FREE SUGARS | | | |
| >5TE% | 0.88 (0.78-0.99) | 0.57 (0.37-0.88) | 0.62 (0.41-0.96) |
| <5TE% |  |  |  |
| >10TE% | 0.85 (0.74-0.97) | 0.77 (0.44-1.36) | 0.86 (0.49-1.53) |
| <10TE% |  |  |  |
| >15TE% | 1.09 (0.92-1.30) | -- | -- |
| <15TE% |  |  |  |
| LIQUID SOURCES OF FREE SUGARS | | | |
| >5TE% | 1.09 (0.95-1.25) | 0.97 (0.63-1.51) | 1.01 (0.65-1.58) |
| <5TE% |  |  |  |
| >10TE% | 1.17 (1.02-1.34) | 1.18 (0.73-1.91) | 1.21 (0.74-1.98) |
| <10TE% |  |  |  |
| >15TE% | 1.43 (1.23-1.66) | -- | -- |
| <15TE% |  |  |  |

^1^ Each threshold of free sugar intake (5TE%, 10TE%) was included in a separate regression model. Associations between GDM and free sugar intake at the 15TE% threshold were not examined due to inadequate statistical power at this level of intake. We compared those with intake above each of these set thresholds to individuals consuming below each respective threshold (reference group). Regression models were adjusted for age, ethnicity, immigrant status, food insecurity, rural residence, smoking, physical activity, total energy intake, amount of food intake reported in the last 24 hours compared to usual intake, consumption of fruit juice daily and ≥5 servings of fruit and vegetables and other dietary covariates (e.g. fats, protein, sodium, potassium, fibre and non-sugar carbohydrates).

**SUPPLEMENTAL TABLE 5** Multivariate associations between baseline characteristics with overweight and with GDM case status

|  |  | Delivery between 2004-2017 |
| --- | --- | --- |
|  | **Baseline associations with overweight (N=6305)** | **Associations with GDM case status**  **(N=1842)** |
| Baseline characteristics^1^ | **Adjusted odds ratio (95% CI)** | **Adjusted odds ratio (95% CI)** |
| Overweight^1^ | -- | 2.58 (1.71 to 3.90) |
| Age, years | 1.05 (1.04 to 1.05) | 1.01 (0.98 to 1.04) |
| Caucasian^2^ | 0.90 (0.75 to 1.08) | 0.61 (0.36 to 1.03) |
| Immigrant^2^ | 0.49 (0.34 to 0.62) | 1.68 (0.80 to 3.52) |
| Food insecure^2^ | 1.61 (1.32 to 1.95) | 1.06 (0.60 to 1.87) |
| Rural residence^2^ | 1.12 (0.97 to 1.27) | 0.92 (0.54 to 1.56) |
| Active^2^ | 0.78 (0.70 to 0.87) | 0.94 (0.62 to 1.43) |
| Smoking^2^ | 1.02 (0.89 to 1.17) | 1.05 (0.66 to 1.69) |
| Energy intake (per 100 kcal) | 1.00 (0.98 to 1.01) | 1.00 (1.00 to 1.00) |
| Amount of food intake reported in last 24-hour compared to usual intake (reference: typical intake) | Much more: 1.15 (0.95-1.39)  Much less: 0.89 (0.77-1.03) | Much more: 1.08 (0.98-1.41)  Much less: 0.72 (0.70-1.01) |
| Non-sugar carbohydrates | 1.00 (0.99-1.01) | 1.01 (0.99-1.04) |
| Saturated fat, TE% | 0.97 (0.95 to 0.99) | 1.03 (0.97 to 1.09) |
| Monounsaturated fat intake, TE% | 1.02 (1.00 to 1.04) | 0.99 (0.93 to 1.06) |
| Polyunsaturated fat intake, TE% | 0.98 (0.95 to 1.01) | 1.02 (0.93 to 1.12) |
| Protein, %TE | 1.02 (1.00 to 1.03) | 1.03 (1.00 to 1.07) |
| Sodium intake, g | 1.04 (1.01 to 1.08) | 1.01 (0.87 to 1.14) |
| Potassium intake, g | 0.95 (0.91 to 0.99) | 0.93 (0.77 to 1.13) |
| Fibre, g | 0.99 (0.98 to 1.00) | 1.00 (0.97 to 1.03) |
| Daily fruit juice^2^ | 0.79 (0.70 to 0.89) | 1.32 (0.87 to 2.01) |
| Fruits/vegetables ≥5 servings/day^2^ | 0.97 (0.84 to 1.10) | 0.68 (0.40 to 1.17) |

^1^The multivariable model examined associations with baseline variables at the above vs. below 5TE% threshold of free sugar intake from solid and liquid sources. Overweight in adolescents (12-19 years old) was defined as corresponding to BMI above the 85^th^ percentile. BMI percentiles were age- and sex- standardized in accordance with Center for Disease Control and Prevention (CDC). Adults were classified as overweight at a BMI equal to or above 25 kg/m^2^.

^2^Odds ratios for dichotomous variables (e.g. Caucasian vs. non-Caucasian, immigrant vs. non-immigrant, etc.). The reference group for each dichotomous variable is represented by the opposing category (e.g. non-Caucasian).

**SUPPLEMENTAL FIGURE 1** Percent of all participants above various thresholds of free sugar (FS) intake as a percent of total energy (TE%)^1^

>2.5TE%

>5TE%

>10TE%

>15TE%

>15TE%

>10TE%

>5TE%

>2.5TE%

**FS IN LIQUIDS**

% of participants

**FS IN SOLIDS**

^1^Overall distribution of free sugar intake from cross-sectional study. The percent of participants above various thresholds by outcome status are reported in Table 2.

**SUPPLEMENTAL FIGURE 2** Percent of delivery cohort above various thresholds of free sugar (FS) intake as a percent of total energy (TE%)^1^

>5TE%

>2.5TE%

>5TE%

>10TE%

>2.5TE%

>10TE%

% of participants

**FS IN LIQUIDS**

**FS IN SOLIDS**

^1^Overall distribution of free sugar intake from nested case-control study. The percent of participants above various thresholds by outcome status are reported in Table 3.

**SUPPLEMENTAL FIGURE 3** Top sources of free sugars from solid sources

**Solid sources**

**(%FS:TS)***

**Food item (%FS:TS)^1^**

^1^FS:TS = free sugars as a percent of total sugars

**SUPPLEMENTAL FIGURE 4** Top sources of free sugars from liquid sources

**Beverage or liquid item (%FS:TS)^1^ (%FS:TS)***

**Liquid sources**

**(%FS:TS)***

^1^FS:TS = free sugars as a percent of total sugars
